# Population history provides foundational knowledge for utilizing and developing native plant restoration materials

**DOI:** 10.1111/eva.12704

**Published:** 2018-09-24

**Authors:** Rob Massatti, Holly R. Prendeville, Steve Larson, Bryce A. Richardson, Blair Waldron, Francis F. Kilkenny

**Affiliations:** ^1^ Southwest Biological Science Center U.S. Geological Survey Flagstaff Arizona; ^2^ U.S.D.A. Forest Service Pacific Northwest Research Station Corvallis Oregon; ^3^ U.S.D.A. Agricultural Research Service Logan Utah; ^4^ U.S.D.A. Forest Service Rocky Mountain Research Station Provo Utah; ^5^ U.S.D.A. Forest Service Rocky Mountain Research Station Boise Idaho

**Keywords:** bluebunch wheatgrass, commercial germplasm, cultivar, fastsimcoal, Great Basin, Intermountain West, Last Glacial Maximum, *Pseudoroegneria spicata*

## Abstract

A species’ population structure and history are critical pieces of information that can help guide the use of available native plant materials in restoration treatments and decide what new native plant materials should be developed to meet future restoration needs. In the western United States, *Pseudoroegneria spicata* (bluebunch wheatgrass; Poaceae) is an important component of grassland and shrubland plant communities and commonly used for restoration due to its drought resistance and competitiveness with exotic weeds. We used next‐generation sequencing data to investigate the processes that shaped *P. spicata*'s geographic pattern of genetic variation across the Intermountain West. *Pseudoroegneria spicata*'s genetic diversity is partitioned into populations that likely differentiated since the Last Glacial Maximum. Adjacent populations display varying magnitudes of historical gene flow, with migration rates ranging from multiple migrants per generation to multiple generations per migrant. When considering the commercial germplasm sources available for restoration, genetic identities remain representative of the wildland localities from which germplasm sources were originally developed, and they maintain high levels of heterozygosity and nucleotide diversity. However, the commercial germplasm sources represent a small fraction of the overall genetic diversity of *P. spicata* in the Intermountain West. Given the low migration rates and long divergence times between some pairs of *P. spicata* populations, using commercial germplasm sources could facilitate undesirable restoration outcomes when used in certain geographic areas, even if the environment in which the commercial materials thrive is similar to that of the restoration site. As such, population structure and history can be used to provide guidance on what geographic areas may need additional native plant materials so that restoration efforts support species and community resilience and improve outcomes.

## 1 INTRODUCTION

Ecosystem disturbances are increasingly common due to natural events (e.g., Balaguru, Foltz, & Leung, 2018; Wotton, Nock, & Flannigan, 2010) and human‐induced activities (Foley et al., 2005). In response, more resources are being devoted to the development and use of native plant materials (e.g., Basey, Fant, & Kramer, 2015; Erickson, 2008; Tischew, Youtie, Kirmer, & Shaw, 2011; Wood, Doherty, & Padgett, 2015), with the hope that restoration using native plant materials can help address specific environmental challenges, rejuvenate ecosystem function, and improve the delivery of ecosystem services (Hughes, Inouye, Johnson, Underwood, & Vellend, 2008). Concurrent research has focused on ensuring that native plant materials are “appropriate” for restoration sites (see, e.g., guidance provided by Plant Conservation Alliance 2015, as well as McKay, Christian, Harrison, & Rice, 2005; Broadhurst et al., 2008; Havens et al., 2015). From a genetic perspective, appropriate native plant materials are those that avoid (or mitigate) risks associated with the mixing of local and nonlocal genotypes (Vander Mijnsbrugge, Bischoff, & Smith, 2010). For example, nonlocal genotypes may not be adapted to the local environment at a restoration site and therefore have lower fitness (Bischoff, Vonlanthen, Steinger, & Muller‐Scharer, 2006; Jones, Hayes, & Sackville Hamilton, 2001; Knight & Miller, 2004; Oduor, Leimu, & van Kleunen, 2016). In addition, nonlocal genotypes are increasingly being implicated in negatively impacting local plant and animal species (Bucharova et al., 2016; Keller, Kollman, & Edwards, 1999; Sinclair et al., 2015; Smith, 2007; Vandegehuchte, De La Pena, Breyne, & Bonte, 2012). Furthermore, the intraspecific hybridization of local and nonlocal genotypes could result in outbreeding depression due to the introgression of maladapted genes or hybrid breakdown (Edmands, 2007; Hufford & Mazer, 2003), or nonlocal genotypes may prove to be better adapted to local ones and become invasive (Saltonstall, 2002); however, the importance of these latter phenomena is debatable based on available evidence (e.g., see Whitlock et al., 2013). Regardless of the potential intraspecific or interspecific impacts resulting from using nonlocal genotypes in restoration treatments, genetic diversity has been recognized as a unit of conservation concern (see Hoban et al., 2013 and references therein), suggesting the maintenance of geographic patterns of genetic variation by avoiding the mixture of local and nonlocal genotypes should be an implicit restoration goal (Bucharova et al., 2018). Therefore, gathering information on the genetics of native plants important to restoration is imperative for making the appropriate seed sourcing decisions for ecosystem restoration (Breed et al., 2018).

Given the potentially negative impacts of using inappropriate native plant materials, multiple approaches have been developed to spatially guide their transfer (i.e., seed transfer zones). At a coarse scale, Bower, St. Clair, and Erickson (2014) created 64 provisional seed zones for the continental United States using biologically important climatic data, as well as regional ecological categorizations (i.e., Omernik level III ecoregions; Omernik, 1987). However, these zones are not species‐specific, and regionally important environmental gradients may not be incorporated due to the continental scale of their analysis (e.g., the monsoonal precipitation gradient across the Colorado Plateau is not represented, but important to species across this region). A species‐specific approach using distribution data (e.g., from vetted herbarium records) and a broader suite of environmental data was developed by Doherty, Butterfield, and Wood (2017); this approach more closely captures and partitions the environmental space occupied by a species to inform seed transfer. Genecological studies that combine phenotypic trait data, as informed by common gardens and/or reciprocal transplants, and climate data have resulted in the inference of seed transfer zones for a variety of species across the western United States (summarized in Kilkenny, 2015). Finally, correlating adaptive genetic variation, as inferred from outlier loci, to climate data can help deduce environmental gradients important to species, thus assist the development of seed transfer zones (Shryock et al., 2017). These latter two approaches are the most informative with respect to the transfer of native plant materials because they resolve species‐specific adaptation to environmental gradients. While all of these approaches may alleviate the potential problems of nonlocal native plant materials at a restoration site, they only tangentially address (if at all) how plants across their distributions are related to one another from an evolutionary perspective. An evolutionary perspective benefits restoration because it reveals the genotypic suitability of native plant materials for a restoration site based on the relatedness of the materials with local conspecifics.

Originating from mutation, genetic variation becomes structured by gene flow, recombination, random genetic drift, and natural selection (Hartl & Clark, 2006). Thus, contemporary population structure reflects the historical events that caused a species’ populations to merge, split, shrink, expand, establish, and disappear. For many organisms around the world, and especially those distributed across higher latitudes in temperate and boreal climates, cyclical glaciations during the Pleistocene were a dominant force influencing population structure (Provan & Bennett, 2008; Shafer, Cullingham, Cote, & Coltman, 2010; Soltis, Morris, McLachlan, Manos, & Soltis, 2006), as fluctuating climates forced many species to track suitable habitat to persist (Avise, 2000). The last glacial period began to recede approximately 20,000 years ago, intimating that organisms in habitats affected by glaciations have occupied their contemporary distributions for fewer than 20,000 years. Furthermore, organisms’ current distributions may result from one or more historically connected or isolated glacial‐age populations (Lanier, Massatti, He, Olson, & Knowles, 2015; Satler & Carstens, 2017). Given that historical connectivity may have profound impacts on contemporary gene flow (Edmands, 2007; Frankham et al., 2011), defining population structure and how those populations have interacted in the past should be of utmost importance when determining a local versus nonlocal genotype. This is underscored by the fact that individuals within a species can share phenotypic traits that are putatively adapted to a specific climate space yet have independent evolutionary histories, such that crossing individuals from these localities may produce unfit hybrids because of the breakdown of coadapted gene complexes (McKay et al., 2005). Evolutionary histories are not usually considered when constructing seed transfer zones (although see Bucharova et al., 2018 for an example of indirect consideration).

To demonstrate the utility of resolving population structure and history to restoration practices, we investigate the genetic patterns of *Pseudoroegneria spicata* (Pursh) Á. Löve (Poaceae; commonly referred to as bluebunch wheatgrass, synonym includes *Elymus spicatus* (Pursh) Gould). This drought resistant, perennial bunchgrass competes well with exotic weeds (Zlatnik, 1999) and is an important member of grassland and shrubland plant communities across the Intermountain West of the western United States. Native perennial grasses such as *P. spicata* are widely utilized for fire rehabilitation and other large‐scale restoration projects across the Intermountain West, where fire cycles influenced by exotic annual grasses have degraded millions of hectares of sagebrush (*Artemisia* L.) steppe (Davies & Johnson, 2017). Several natural‐origin *P. spicata* germplasm sources (hereafter referred to as commercial germplasm sources) have been developed from the Palouse region of Washington (Jones et al., 2002; Larson, Jones, Hu, McCracken, & Palazzo, 2000; Ogle, St. John, & Jones, 2010). In addition, seed transfer zones have been delineated for *P. spicata* in the Intermountain West to guide the deployment of native plant materials (St. Clair, Kilkenny, Johnson, Shaw, & Weaver, 2013).

Here, we use a next‐generation sequencing dataset developed for *P. spicata* to elucidate the dynamics of *P. spicata* populations through time and the genetic relationships of the available commercial germplasm sources to regional wildland localities, with the goal of providing information relevant to the use of available native plant materials and the future development of additional native plant materials. We first describe *P. spicata*'s population structure across the Intermountain West. Given *P. spicata*'s large geographic distribution over a topographically diverse landscape, it is unknown whether contemporary populations persisted in one refugium or multiple refugia during the last glacial period, or how frequent historical gene flow among populations may have been. Therefore, we use our population structure results to inform coalescent modeling scenarios to estimate divergence times and migration rates between adjacent populations. Our analyses include *P. spicata* commercial germplasm sources that are commonly used in restoration to facilitate comparison with wildland populations, as well as characterization of genome size and ploidy level, which varies across *P. spicata*'s distribution (Gibson, Fishman, & Nelson, 2017) and can have significant effects on restoration treatments (Kramer, Wood, Frischie, & Havens, 2018). This research strategy is widely applicable as more native plant materials are generated and used in restoration and conservation projects (Plant Conservation Alliance, 2015).

## 2 METHODS

### 2.1 Field sampling and DNA extraction

**Figure 1:**
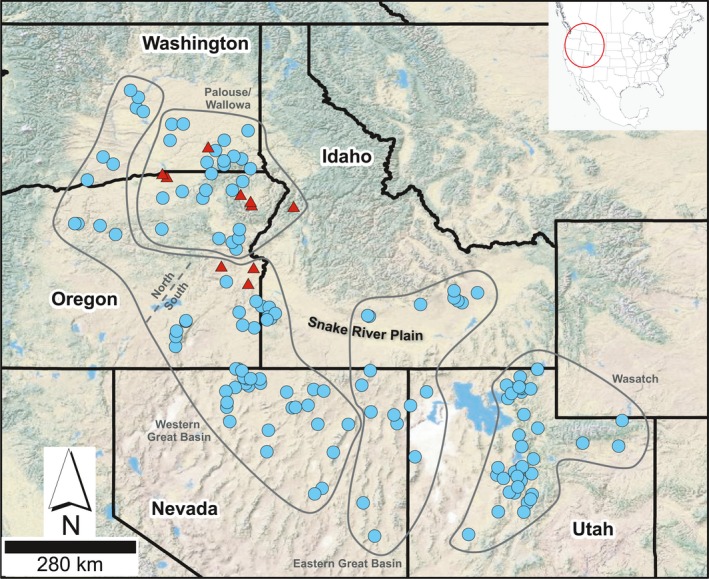
Sampling localities across western North America. Symbols denote sampling localities containing only *Pseudoroegneria spicata* (blue circles) and localities containing *Elymus wawawaiensis* individuals (red triangles). Polygons delineate genetic clusters referred to in the text and include Wasatch (WAS), Eastern Great Basin (EGB), Western Great Basin (WGB), and Palouse/Wallowa (P/W)

*Pseudoroegneria spicata* was sampled throughout the Intermountain West during multiple collection efforts (Figure 1). At each of 154 wildland sampling localities (Supporting Information Table S1 and Figure 1), reproductive stalks were collected from 60 to 500 individuals distributed across 0.5–5 acres. Seeds were pooled by sampling locality, cleaned to remove chaff, and stored in airtight containers in a refrigerated room. From these collections, random samples of seed were germinated and grown in a greenhouse for leaf tissue to use in DNA extraction. These efforts resulted in 887 unique individuals from localities distributed across five western states (average of 5.8 individuals per site, see Supporting Information Table S1).

In addition to wildland‐collected seed, we obtained seed from eight commercial germplasm sources that are commonly used in restoration treatments. Leaf material was generated as described above and sampled for DNA extraction (10 individuals per commercial germplasm source). Six of the commercial germplasm sources represent *P. spicata* (Anatone, Columbia, Goldar, P‐7, Wahluke, and Whitmar). The final two commercial germplasm sources, Secar and Discovery, represent *Elymus wawawaiensis* J.R. Carlson & Barkworth, which is an allotetraploid containing copies of the St (*Pseudoroegneria*) and H (*Hordeum*) genomes (Carlson & Barkworth, 1997; Mason‐Gamer, 2001; Mott et al., 2011). *Elymus wawawaiensis* is codistributed with *P. spicata* throughout part of *P. spicata*'s distribution and separating the taxa can be difficult due to similar morphological characteristics. As such, inclusion of Secar and Discovery individuals allowed us to identify and exclude any *E. wawawaiensis* individuals in our dataset.

Approximately 15 mg of leaf tissue was ground using a bead mill, and DNA was extracted following the DNeasy plant extraction kit protocol (Qiagen, Germantown, MD, USA). Genome size and ploidy were determined for wildland‐collected individuals using a Partec Cyflow Space flow cytometer under UV fluorescence with an *Atriplex canescens* (Pursh) Nutt. internal standard. The sample and standard were finely chopped, and nuclei extraction and staining followed the Cystain UV Precise P assay procedure (Sysmex). Sampling localities were screened for polyploidy by randomly choosing three individuals per locality. If polyploid individuals were identified, an additional three samples were assessed to estimate the proportion of polyploids at the sampling locality. While using only three individuals per sampling locality for the primary screening may have caused us to miss polyploids when present in low frequencies, we note that this method correctly identified all sampling localities containing allotetraploids (i.e., *E. wawawaiensis*), regardless of their frequency in the population (as assessed using the genomic data—see Results).

### 2.2 Next‐generation sequencing and data processing

A total of 967 individual plants (887 wildland‐collected + 80 individuals from commercial germplasm sources) were selected for genotyping‐by‐sequencing (Elshire et al., 2011; Poland, Brown, Sorrells, & Jannink, 2012). Genomic DNA from individual plants was digested with *Pst*I and *Msp*I, followed by the ligation of Illumina adaptor sequences; each individual was barcoded four times using unique, 5–10 base pair barcodes. Ligation products were pooled, purified using QIAquick PCR kits (Qiagen), and amplified using 16 cycles of PCR with eight replicates. A Pippin Prep (Sage Science, Beverly, MA, USA) was used to size select amplicons from 400 to 500 base pairs. Nine 96‐sample genotyping‐by‐sequencing libraries, with four barcodes for each sample (i.e., 384 unique indexes per library), were sequenced on a HiSeq 2500 (Illumina, San Diego, CA, USA) to generate 1 × 125 base pair reads.

Raw data were demultiplexed, filtered, and assembled de novo using stacks version 1.46 (Catchen, Hohenlohe, Bassham, Amores, & Cresko, 2013). First, we used the *process_radtags* script to exclude raw reads containing more than four low‐quality sites, adapter contamination, and/or ambiguous barcodes. Reads were truncated to 100 base pairs due to variation in barcode lengths, read lengths, and read quality at the end of raw reads. Next, each individual's sequences were clustered into highly similar stacks (i.e., sets of sequences inferred to be from a single locus) with the ustacks program using a minimum stack depth (*m*) of 3 and a distance between stacks (*M*) of 2 (parameter choice was informed by Paris, Stevens, & Catchen, 2017). We constructed a catalog of consensus loci using the cstacks program that contained 34 individuals, and loci were merged across individuals if the distance between them (*n*) was ≤2. The individuals chosen to construct the catalog represent wildland sampling localities across the geographic distribution of our sampling scheme; we limited the sample size to minimize error and restrict the accumulation of loci found in only a few individuals (Catchen et al., 2013). The catalog was used to determine the allele(s) present in each individual at each homologous locus during the execution of the sstacks program.

To create datasets used in analyses, we first executed the populations program in the stacks pipeline using unrestrictive parameters (*p* = 1, *r* = 0) to generate a Variant Call Format (vcf) file. We utilized a custom script to read the vcf file and calculate θ based on the number of segregating sites across loci in r (r Core Team, 2017). Using a 95% quantile cutoff, we identified extremely variable loci (i.e., those that contained an abundance of single nucleotide polymorphisms, or SNPs) and created a “whitelist” (i.e., a list that excluded the excessively variable loci) that was used in a second populations execution with the same parameter values to create a filtered vcf file. The filtered vcf file was further processed with a separate script to exclude all SNPs with >70% missing data across individuals and individuals missing >90% of loci (a threshold which identified a clear outlier group of individuals). After filtering, the script wrote a structure formatted file, which was read into r using the adegenet (Jombart, 2008) read.structure function. Major axes of genetic variation were visualized with principal component analysis (PCA) using the dudi.pca function in r (center = T, scale = T with missing data replaced by the mean frequency of the corresponding allele). By iteratively using PCA and our filtering script, we were able to identify outlier individuals (i.e., individuals displaying unique genetic identities resulting from unknown processes, but likely an artifact of library construction or sequencing idiosyncrasies) and assess the impact of various amounts of missing data, thereby maximizing the number of SNPs while minimizing the loss of information content (for further details, see Massatti, Doherty, & Wood, 2018).

Population genetics statistics were calculated for diploid *P. spicata* sampling localities and commercial germplasm sources using an independent iteration of populations (*p* = 2, *r* = 0.50). We assessed the impact of variation in the number of individuals per sampling locality/commercial germplasm source by including all individuals as well as subsampling down to four individuals per sampling locality/commercial germplasm source, which represented the smallest number of sampled individuals; subsampling and statistic calculation were performed 20 independent times. A final execution of populations only included sampling localities pertaining to fastsimcoal2 modeling (see below) and generated a phylip file, which was analyzed with PhyML (Guindon et al., 2010; Lefort, Longueville, & Gascuel, 2017), and fixation index (*F*_ST_) statistics; together, these data informed the fastsimcoal2 modeling scenarios.

### 2.3 Population structure

We used two approaches to infer genetic structure within *P. spicata*: (a) Bayesian clustering implemented in structure version 2.3.4 (Falush, Stephens, & Pritchard, 2003; Pritchard, Stephens, & Donnelly, 2000) and (b) a multivariate ordination method that accounts for spatial patterns, spatial principal component analysis (sPCA), implemented in the adegenet package (Jombart, 2008) in r. We used both methods to allow comparison of results across approaches with different sets of assumptions. For example, structure assumes that loci are in equilibrium and unlinked while sPCA does not. Furthermore, Bayesian clustering may be inappropriate when populations are structured across a gradient of introgression (Jombart, Devillard, Dufour, & Pontier, 2008) because it may overestimate genetic structure, while a spatially explicit multivariate method can identify genetic structure, including clines, and accounts for spatial autocorrelation (Frantz, Cellina, Krier, Schley, & Burke, 2009). structure was run across *K*‐values ranging from 1 to 10 without assigning population membership a priori. Twenty independent runs per *K* were conducted, each with 150,000 burn‐in and 500,000 Markov chain Monte Carlo iterations, using an admixture model with correlated allele frequencies. structure harvester (Earl & VonHoldt, 2012) and distruct (Rosenberg, 2004) were used to visualize results, and the most probable *K* was chosen based on Δ*K* (Evanno, Regnaut, & Goudet, 2005). For sPCA analysis, geographic locations of individuals were created by jittering the latitude/longitude of their sampling localities (factor = 3), and a Delauney triangulation graph was used to create the connection network required by the sPCA function.

### 2.4 Estimating population divergence, population size, and gene flow

**Figure 2:**
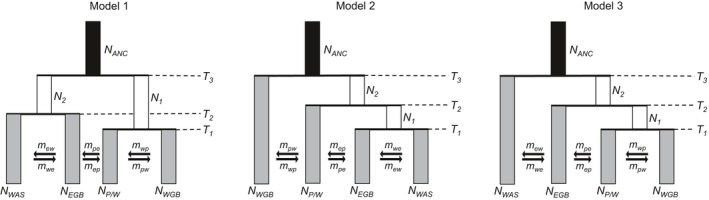
Schematics of fastsimcoal2 modeling scenarios. All italicized labels represent estimated parameters, except for the ancestral effective population size (*N*_ANC_), which was set to 60,000 (see Methods). The rate of migration (*m*) is assumed to be directionally the same between population pairs. Each model has the same parameter values, though they vary in their order and/or representation. To crosswalk to *K *= 4 in Figure 4: *N*_WAS_ **= Wasatch sampling localities (red); *N*_EGB_ **= Eastern Great Basin (yellow); *N*_P/W_ **= Palouse/Wallowa (orange); and *N*_WGB_ **= Western Great Basin (blue)

In order to investigate the impact of Pleistocene glaciations on *P. spicata*, parameters including population divergence time (*T*), population size (*N*_*e*_), and gene flow (2Nm) were estimated from the SNP data using an allele frequency spectrum method (Gutenkunst, Hernandez, Williamson, & Bustamante, 2009) implemented in fastsimcoal2 (version 2603; Excoffier, Dupanloup, Huerta‐Sánchez, Sousa, & Foll, 2013). This procedure uses coalescent simulations to calculate the likelihoods of observed allele frequency spectra (see Nielsen, 2000) under user‐specified demographic models. Because the true relationships of *P. spicata* populations across the West are unknown, we estimated parameters under three 4‐population models that were constructed using patterns resolved from population structure and phylogenetic analyses (Figure 2). We did not include all sampled individuals in our modeling efforts, but only those from localities most representative of the major genetic axes (i.e., see the most differentiated populations represented by spatial principal component 2 and spatial principal component 3 in Supporting Information Figure S3, as well as Supporting Information Table S1 for population assignments). Allowing for multiple population histories allowed us to select the best‐supported model using Akaike information criterion (Akaike, 1974).

A folded joint site frequency spectrum (i.e., for the minor allele, in the absence of information for the derived state) was calculated for each population pair based on polymorphic loci recorded in the whitelist‐filtered vcf file containing only *P. spicata*. One SNP per locus was randomly chosen to satisfy the fastsimcoal2 likelihood assumption that SNPs are in linkage equilibrium. To remove all missing data for the calculation of the joint site frequency spectrum, each population was subsampled using easySFS.py (https://github.com/isaacovercast/easySFS) and only loci found in at least 13, 13, 24, and 22 individuals in the Wasatch, East Great Basin (EGB), Palouse/Wallowa, and West Great Basin (WGB) populations (see Results for population definitions), respectively, were retained to minimize errors with allele frequency estimates. Numbers of individuals per population were determined based on the number of individuals and number of loci in each of the groups.

Divergence times (values of *T*) were estimated accounting for the possibility of migration (values of *m*) and variation in effective population sizes (values of *N*) (Figure 2). To improve the performance of the models by reducing the number of estimated parameters (Excoffier et al., 2013), one population parameter was calculated directly from the data. Specifically, the ancestral effective population size (*N*_ANC_) was fixed, whereas the other parameters were estimated based on the site frequency spectrum (see Figure 2). The ancestral effective population size was calculated using the equation: *θ*_π_/2/(mutation rate × generation time), assuming a genomewide SNP mutation rate similar to *Arabidopsis thaliana* (7 × 10^−9^ per site per generation; Ossowski et al., 2012) and a generation time of 1 year (i.e., the time to first potential reproduction for a newly established plant). One hundred runs per model were conducted and the global maximum likelihood solution is presented. Each run was performed with 200,000 simulations per likelihood estimation and 40 expectation‐conditional maximization (ECM) cycles. Parameter confidence intervals were calculated from 100 parametric bootstrap replicates, by simulating site frequency spectra with the same number of SNPs from the maximum composite likelihood estimates and re‐estimating parameters each time (Excoffier et al., 2013).

## 3 RESULTS

### 3.1 NGS data quality, processing, and dataset construction

Nine lanes of Illumina sequencing produced >2.5 billion reads across 967 individuals (average of 2.6 × 10^6^ per individual), of which >940 million passed quality control (Supporting Information Table S1). Based on low coverage or low quality of reads, eight individuals were excluded from further analyses. After investigating a range values for missing data across SNPs, we determined that a 20% threshold represented the best balance between minimizing potentially negative impacts of missing data on inference and minimizing the loss of information content (signal). Under this threshold, datasets included: a PCA dataset containing all individuals (*P. spicata *+ *E. wawawaiensis*: 959 individuals, 8945 unlinked SNPs); a PCA dataset including only *P. spicata* (834 individuals, 3,040 unlinked SNPs), which was filtered down to 776 individuals (e.g., excluding individuals representing the commercial germplasm sources) and used in structure and sPCA. The vcf file from which the fastsimcoal2 site frequency spectrum was calculated included 295 individuals and 113 310 genotyping‐by‐sequencing loci; this dataset was also used to calculate corrected AMOVA *F*_ST_ values and generate a phylip file with 213,746 SNPs for the four populations resolved by structure and sPCA. Diversity statistics were calculated in populations from a vcf file containing 834 individuals and 113,310 loci. Data sets are archived in the Dryad Digital Repository (https://doi.org/10.5061/dryad.rc1jr0v).

### 3.2 Genetic differentiation, diversity, and structure

An initial PCA on the full dataset clearly differentiated *E. wawawaiensis* (i.e., the Secar and Discovery commercial germplasm sources) from *P. spicata* individuals along principal component 1, while variation within *P. spicata* was described by principal component 2 (Supporting Information Figure S1). We identified and excluded from further analyses 66 individuals from wildland sampling localities that clustered with (or near) *E. wawawaiensis* individuals (Supporting Information Table S1). The identification of mixed‐ploidy or tetraploid‐only sampling localities using genetic data precisely matched ploidy estimations for sampling localities made using flow cytometry, in terms of *E. wawawaiensis*. Flow cytometry identified two additional sampling localities as containing polyploids, but these localities did not contain *E. wawawaiensis* according to the PCA. Presumably, these sampling localities contained autotetraploid individuals (Carlson & Barkworth, 1997; Gibson et al., 2017), and they were excluded from further analyses because stacks and other analytical methods assume diploidy (see Supporting Information Table S1).

**Figure 3:**
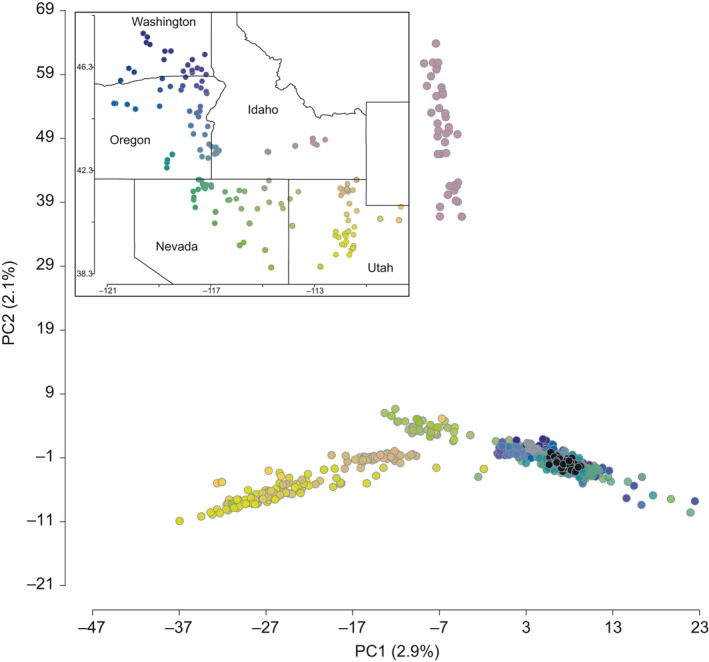
Distribution of diploid *Pseudoroegneria spicata* individuals along principal component 1 and principal component 2 axes of genetic variation. Variation explained by the axes is given in parentheses. Individuals in PC space are colored by their sampling locations, which are displayed in the inset (see also Figure 1). Individuals colored black represent the commercial germplasm sources. Colors in this figure do not correlate to colors that describe populations (i.e., Figures 4 and 5)

**Figure 4:**
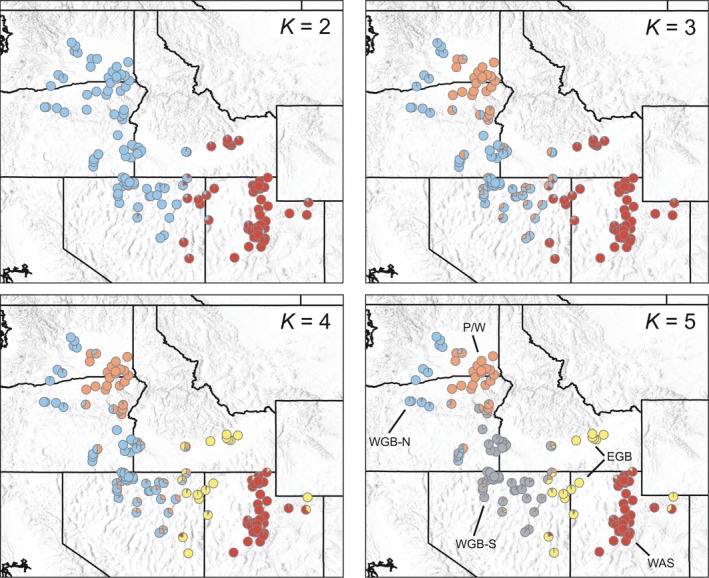
Results of structure analyses for *K *= 2 to *K *= 5. The posterior probabilities of individual assignments are averaged within sampling localities and represented by pie charts. Populations discussed in the text are noted for *K *= 5 and include Palouse/Wallowa (P/W); Western Great Basin (WGB‐North and WGB‐South); Eastern Great Basin (EGB); and Wasatch (WAS)

The PCA on diploid *P. spicata* individuals describes major geographic groups across our sampling area (Figure 3). Principal component 1 is positively correlated with east‐to‐west variation (i.e., Wasatch Mountains in Utah to Oregon and Washington). Principal component 2 is dominated by variation predominantly sampled from localities on the eastern half of the Snake River Plain in Idaho. In general, individuals from sampling localities cluster with one another and close to individuals from geographically proximate localities (Figure 3). Individuals representing commercial germplasm sources form a tight cluster that overlaps individuals from Oregon and Washington (Figure 3). Upon closer examination, commercial germplasm sources cluster with (i.e., are most genetically similar to) the wildland sampling localities closest to where the original foundational materials were collected (for the respective commercial germplasm source; as determined using Ogle et al., 2010).

structure analyses indicate *K *=* *5 as the most likely number of genetic clusters (the *K‐*value with the highest Δ*K*, hereafter referred to as the most likely *K*). We present illustrations of *K *=* *2–5, as they are all helpful in unpacking the major, hierarchical axes of genetic variation within *P. spicata* (Figure 4). At *K *=* *2, genetic variation breaks down into northwestern (blue) and southeastern (red) genetic clusters. An orange genetic cluster centered in southeastern Washington to northeastern Oregon is separated from the blue cluster at *K *=* *3, and a yellow genetic cluster located primarily in the eastern Snake River Plain and eastern Nevada splits from the red cluster at *K *=* *4. Finally, the blue genetic cluster decomposes again at *K *=* *5, resulting in a southern gray cluster. In general, admixture is more common where genetic clusters meet, compared to the “cores” of their respective geographic distributions. These structure results contrast with the 21 clusters of variation reported by Larson, Jones, and Jensen (2004), which were based on a model selected solely from the log probability of data resolved by structure without consideration of model complexity, as suggested by Evanno et al. (2005). Despite the differences between these studies, Larson et al. (2004) break down hierarchical variation into finer units we focus on here. While it is likely our genetic clusters would hierarchically decompose if analyzed in isolation (e.g., Massatti & Knowles, 2014; Ryan, Bloomer, Moloney, Grant, & Delport, 2007), this level of detail is not necessary for the questions at hand.

The first three sPCA eigenvalues associated with global structure were used to characterize genetic variation among *P. spicata* sampling localities (Supporting Information Figure S2). In general, patterns among spatial principal components 1–3, which explain 49.4% of genetic variation, reiterate the clusters resolved by structure analyses. Spatial principal component 1 identifies the northwestern/southeastern grouping similar to *K *=* *2, and spatial principal component 2 resolves differentiation between the southeastern Washington to northeastern Oregon sampling localities and localities surrounding this area to the west and south (akin to *K *=* *3; Supporting Information Figure S3). Finally, spatial principal component 3 differentiates sampling localities from the eastern half of the Snake River Plain and eastern Nevada from the rest (i.e., *K *=* *4). Unlike structure analyses, where the blue genetic cluster breaks down from *K *=* *4 to *K *=* *5, sPCA does not resolve this as a significant genetic axis. Because sPCA accounts for geographic distance among sampling localities when identifying global and local structures, we hypothesize that *K *=* *5 represents isolation by distance along a latitudinal cline of the blue genetic cluster identified at *K *=* *4 (Figure 4) (see also Supporting Information Figure S4 for a RGB composite illustration of genetic similarity, which shows a continuous grade from the blue to gray genetic clusters). Hereafter, we focus on the genetic clusters (which we call “populations”) identified at *K *=* *4. Furthermore, we assign these populations the following names, which are reiterated in Figure 4: red genetic cluster—Wasatch (WAS); yellow cluster—EGB; orange cluster—Palouse/Wallowa (P/W); and blue cluster—WGB. We further specify WGB‐North and WGB‐South to recognize the north/south differentiation in this population (i.e., the blue and gray clusters, respectively) discerned in the *K *=* *5 structure result.

The relationships among the four populations are supported by the hierarchical decomposition of the genetic variation from *K *=* *2 to *K *=* *5 (Figure 4) and from spatial principal components 1–3 (Supporting Information Figure S3), as well as by *F*_ST_ values and the maximum likelihood tree reconstruction. P/W and WGB are the most similar populations, as inferred from the lowest *F*_ST_ value (Table 1) and the short branches in the maximum likelihood tree (Supporting Information Figure S5). Each of these populations is differentiated from EGB and WAS as would be expected based on geographic distance (Table 1). While WAS and EGB are highly supported as sister populations (Supporting Information Figure S5), they are also the most differentiated (Table 1). Given that the highest levels of hierarchical variation (i.e., *K *=* *2 and spatial principal component 1) group WAS and EGB sampling localities together, these results justify our choice of alternative models used for fastsimcoal2 model selection and parameter estimation, which include the relationships proposed by the maximum likelihood tree (Model 1) and two isolation by distance scenarios (Models 2 and 3; Figure 2).

**Table 1:** Pairwise corrected AMOVA *F*_ST_ calculated between the populations used in fastsimcoal2 modeling. All values are significant at *p *<* *0.05. See Table S1 to determine which sampling localities are included in each population

	WGB	EGB	WAS
P/W	0.041	0.068	0.075
WGB		0.079	0.085
EGB			0.099

*Note*

P/W: Palouse/Wallowa; WGB: Western Great Bain; EGB: Eastern Great Basin; WAS: Wasatch.

**Figure 5:**
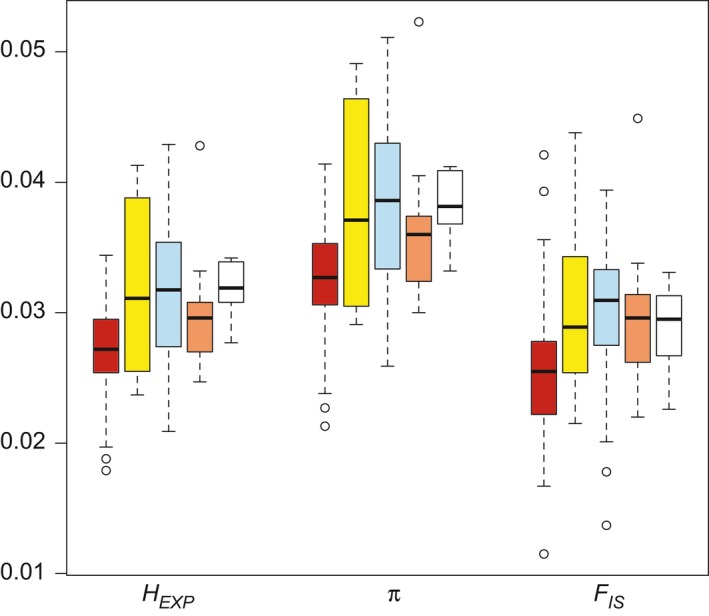
Comparison of genetic diversity statistics across populations identified in the *K *= 4 structure analysis (red—WAS, yellow—EGB, blue—WGB, and orange—P/W; see Figures 1 and 4 for population definitions). The white box‐and‐whisker plots represent the commercial germplasm sources. For each summary statistic (*H*_EXP,_ expected heterozygosity; π, nucleotide diversity; *F*_IS_, inbreeding coefficient), the median, first and third quantiles, standard deviation, and range across populations are shown

Genetic diversity statistics were similar across the *P. spicata* populations (Figure 5 and Supporting Information Table S2). The eastern and western populations (as defined by *K *=* *2 in Figure 4) have similar ranges of expected heterozygosities (*H*_EXP_), nucleotide diversities (π), and inbreeding coefficients (*F*_IS_) (i.e., when considering WAS + EGB vs. P/W  +  WGB), and both have a genetic cluster that has, on average, lower diversity (i.e., WAS and P/W), and a cluster with a greater range of diversity (i.e., the EGB and WGB). The EGB genetic cluster in Figure 5 can be broken into two groups: one group containing the eastern Snake River Plain sampling localities, which have genetic diversity statistics generally above the median expected heterozygosity and nucleotide diversity; and a second group containing eastern Nevada and northwestern Utah localities, which have genetic diversity statistics generally below the median expected heterozygosity and nucleotide diversity (Figure 5 and Supporting Information Table S2). Subsampling to four individuals per sampling locality had no effect on the distribution of diversity statistics across the populations, and we present only statistics calculated on the full dataset (i.e., Figure 5 and Supporting Information Table S2).

### 3.3 Demographic parameter estimation

The *P. spicata* population history represented by Model 3 has the lowest Akaike information criterion (148,750) compared to Models 1 and 2 (Akaike information criteria = 149,266 and 149,388, respectively); in other words, Models 1 and 2 minimize information loss negligibly compared to Model 3 (each is 0 times as probable for minimizing loss; Figure 2). Similarly, Model 1 is much more likely and minimizes information loss considerably, compared to Model 2. There are many similarities in parameter estimations under the three alternative population histories (Table 2), and especially between Models 3 and 1. Estimates of present‐day effective population sizes (*N* values) are similar among all three models, including in relative differences among populations, with the WAS (*N*_*WAS*_) and WGB (*N*_*WGB*_) populations inferred as having the largest and smallest effective population sizes, respectively. Migration rates (*m* values) are also similar (relative to one another and in magnitude), with the highest rates inferred between WGB and P/W, and the lowest rates occurring between WAS and the EGB (Table 2). Notable differences between the models are (a) the history of population expansions and contractions during divergence events and (b) the timing of divergence of the ancestral population (*T*_3_)—in Models 1 and 3, divergence coincides roughly with the end of the Last Glacial Maximum, while in Model 2 the divergence of the ancestral population is near the height of the last glacial period.

**Table 2:** Results of parameter estimation with fastsimcoal2 under three alternative models. Composite maximum likelihood estimates of: effective population sizes (*N*) are presented as the number of individuals per population; divergence times (*T*) are presented as the number of generations (i.e., number of years ago, as 1 generation = 1 year); and migration rates (*m*) are presented as 2Nm. See Figure 2 to determine where each parameter maps onto the respective model. 95% confidence intervals were calculated from 100 parametric bootstrap replicates

Parameter	Model 1		Model 2		Model 3	
	Point estimate	95% CI	Point estimate	95% CI	Point estimate	95% CI
*N*_1_	3,699	3,659–4,258	506,615	485,887–519,774	8,033	7,839–8,405
*N*_2_	288,066	264,045–301,009	116,398	104,249–143,609	3,014	2,698–3,420
*N*_WAS_	53,347	52,773–53,855	49,589	49,020–49,805	62,936	62,546–63,308
*N*_EGB_	34,107	33,867–34,589	37,414	37,070–37,658	45,907	45,597–46,245
*N*_P/W_	21,833	21,504–22,027	37,272	36,979–37,482	30,800	30,698–31,094
*N*_WGB_	11,813	11,559–11,917	6,858	6,832–6,920	10,363	10,257–10,411
*T*_1_	16,007	15,903–16,140	13,835	13,697–13,985	15,211	14,942–15,343
*T*_2_	13,513	13,325–13,619	29,483	29,267–29,682	23,171	23,156–23,501
*T*_3_	21,781	21,265–22,107	30,923	30,638–31,004	24,740	24,559–24,946
*m*_we_	0.03	0.02–0.04	0.13	0.13–0.14	0.46	0.45–0.48
*m*_ew_	0.05	0.03–0.06	0.18	0.17–0.19	0.64	0.62–0.65
*m*_ep_	0.65	0.64–0.66	0.66	0.65–0.66	0.68	0.68–0.70
*m*_pe_	1.02	1.00–1.04	0.67	0.66–0.67	1.02	1.01–1.04
*m*_pw_	4.94	4.89–5.08	4.72	4.66–4.90	4.23	4.17–4.25
*m*_wp_	9.13	9.04–9.39	25.64	25.27–26.61	12.57	12.38–12.63

## 4 DISCUSSION

*Pseudoroegneria spicata* genetic diversity is partitioned across western North America into populations that are robust and repeatable across analyses. Modeling results suggest that these populations have differentiated since the Last Glacial Maximum, and that adjacent populations exchanged migrants at different rates following divergence. This information may significantly contribute to ongoing use of *P. spicata* plant materials in restoration projects, as well as inform the development of new materials.

### 4.1 Population history of *P. spicata*

Pleistocene climatic oscillations had a profound impact on the genetic structure of many organisms in western North America (Carstens, Brunsfeld, Demboski, Good, & Sullivan, 2005; Knowles & Massatti, 2017; Shafer et al., 2010). Especially at higher latitudes in temperate and boreal climates, organisms had opportunities to be separated for prolonged periods of time due to range shifts concordant with climatic oscillations and isolation into allopatric refugia with reduced or absent gene flow (Lanier et al., 2015; Richardson & Meyer, 2012). Alternatively, species presently occupying higher elevation habitat may have had larger, more connected distributions during glacial periods (e.g., Galbreath, Hafner, Zamudio, & Agnew, 2010; Massatti & Knowles, 2016). Population divergence times suggest that *P. spicata* distributed across the landscape was highly affected by glaciations, as genetic variation coalesces to a single, panmictic population roughly concordant with the Last Glacial Maximum (Table 2). Hypothetically, *P. spicata* persisted within a refugium during the last glacial period, and when the climate warmed and deglaciation commenced about 20 KYA, it dispersed into newly suitable habitat to eventually occupy its current distribution. As new locations were occupied and new climates were encountered, neutral and adaptive evolutionary processes facilitated differentiation (Hartl & Clark, 2006).

Determining the location of a refugium is an inherently difficult task (e.g., He, Prado, & Knowles, 2017). Paleovegetation and prehistoric climate data compiled from sites across the West suggest cooler climatic conditions during the Last Glacial Maximum (Grayson, 2006; Ray & Adams, 2001; Thompson & Anderson, 2000), especially in the Great Basin (Waltari & Guralnick, 2009), where *P. spicata* is currently restricted to moderate elevations in mountain ranges. *Pseudoroegneria spicata* grows during cooler parts of the year (i.e., it is a C3 species), and we hypothesize that a cooler climate during the last glacial period may have supported a cohesive *P. spicata* population at lower elevations (compared to its present Great Basin distribution) somewhere in the vicinity of the Central Basin and Range ecoregion (Omernik, 1987). A southerly Great Basin refugium would support the fastsimcoal2 model with the lowest Akaike information criterion (Model 3, or isolation by distance with the earliest divergence being the WAS population)—as the climate warmed and *P. spicata* spread north (and/or east and west), the Wasatch Mountains were colonized early on. As the climate continued to warm, this population became more isolated as lower elevation habitat became inhospitable, while the geographic distribution of the species continued to expand northward. Sampling *P. spicata* throughout its remaining (i.e., unsampled), US distribution would help determine how many refugia persisted during the Last Glacial Maximum and may facilitate better predictions on where the refugia were located.

Dispersal from a common refugium since the Last Glacial Maximum does not preclude long‐term isolation among contemporary populations. Estimates of migration rates indicate that alleles have been exchanged between adjacent populations, although at drastically different rates. While several migrants per generation (on average) have been exchanged between P/W and WGB since their divergence, it has taken multiple generations, on average, for one effective migrant to be exchanged between WAS and EGB (Table 2). Habitat connectivity likely plays a large role in this disparity, as suitable *P. spicata* habitat is separated by inhospitable, lower elevation basins in much of southern Oregon, Nevada, and Utah (e.g., see herbarium voucher records on swbiodiversity.org/seinet/collections). For example, the WAS population is highly differentiated from localities to the west in northwest Utah and eastern Nevada representing the EGB population (Supporting Information Figure S3), a pattern that may be attributed to low habitat suitability across the Bonneville Salt Flats in north‐central Utah. Another steep cline of genetic differentiation exists between the P/W and WGB populations in Oregon and Washington (Supporting Information Figure S4). Here, alternative explanations facilitating differentiation would have to be investigated, because there are no clear areas of low habitat suitability and these populations have regularly exchanged alleles through time (Table 2). Such a steep cline despite high relative levels of gene flow may be indicative of a strong selective regime, perhaps imposed by the precipitation gradient driven by the rain shadow of the Cascade Range (Siler, Roe, & Durran, 2013). While multiple processes likely affect *P. spicata* across its range (i.e., both neutral and adaptive), it is likely that isolated *P. spicata* localities throughout the Intermountain West function to maintain some level of cohesiveness for the species (i.e., a metapopulation framework sensu Hanski, 1998; Supporting Information Figure S4).

Assumptions relied upon in a coalescent modeling framework (e.g., mutation rate and generation time) have the capacity to influence parameter estimations. In general, it is assumed that more data, such as is generated using next‐generation sequencing technologies, should lead to more precise estimates of parameters such as population divergence (Edwards & Beerli, 2000). The influence of assumptions on parameters is exacerbated when relying on external information, though there are rarely better options when working with nonmodel species. Here, we utilized a direct estimate of the genomewide SNP mutation rate from *Arabidopsis thaliana* (Ossowski et al., 2012), and its relevance to species in Poaceae may be suspect because ancestors of these groups likely diverged in the Upper Jurassic (Huang et al., 2016). Perhaps a larger concern is the generation time, which we assumed to be 1 year for *P. spicata*. Within a coalescent modeling framework, generation time represents the time to the first potential reproduction of a plant, and not the lifespan of the plant within the community. When a species within a community reaches carrying capacity, individuals will be replaced, on average, at a rate equivalent to their generation time, regardless of how long an individual can persist. We note that *P. spicata* may have a longer generation time (e.g., 2–3 years) in natural communities. However, our preliminary modeling trials using a range of generation times, as well as independent research (e.g., Satler & Carstens, 2017), suggest that estimated divergence times between populations would be greater when using longer generation times. Such results reinforce our interpretation of how population history would affect the use and development of native plant materials.

### 4.2 Management implications

#### 4.2.1 Applicability of genetic analyses to restoration and conservation efforts

Genetic analyses of genomewide SNP variation yielded information pertinent to restoration efforts. With respect to native plant materials available for restoration, the commercial germplasm sources remain genetically representative of the wildland localities from which plant materials were originally collected (Figure 3). However, the commercial germplasm sources represent a small fraction of the overall genetic diversity of *P. spicata*. Given the close relationship and highest resolved migration rate between the WGB and P/W populations, the risk of unintended consequences (e.g., the negative impacts of nonlocal genotypes on local plant and animal species or outbreeding depression) may be lowest when the commercial germplasm sources are used in restoration treatments across the geographic area covered by these populations. Risks may increase when commercial germplasm sources are used in restoration treatments located within the distribution of the WAS or EGB genetic clusters due to the low levels of gene flow and/or long divergence times between these populations and the P/W and WGB populations from which commercial germplasm sources were developed. As such, future restoration treatments outside of the geographic area covered by P/W and WGB may benefit from the development of plant materials representing the EGB and/or WAS genetic identity. We note that the ultimate test of negative consequences that may be realized by using restoration materials outside of their optimal geographic range (i.e., in areas occupied by highly divergent populations) would be a crossing experiment between the commercial germplasm source and the local plants that would track seed production and the subsequent germination/survival rate of the seedlings.

The collection, development, and deployment of plant materials as suggested by the seed zones of St. Clair et al. (2013) may also be informed by spatial patterns of genetic variation and population history. These seed zones were developed using a genecological approach, which utilized phenotypic data from common gardens and climatic variability across the northwestern United States. Considering the geographic distributions of the populations resolved here in relation to the distribution of the seed zones (see their Figure 3), we note that each of our populations is distributed across several, if not all, of the seed transfer zones. Given that sampling localities within a population are more closely related to one another (in a phylogenetic sense) than they are to localities from another population, we infer that the ancestors of all four populations independently adapted into the environmental space represented by the seed zones. This speaks to the adaptability of wildland populations and supports the idea that managing for genetic diversity should be an important conservation goal (Hoban et al., 2013). In addition, all populations except WAS are distributed across multiple level III ecoregions (Omernik, 1987), and similarly, almost all level III ecoregions contain multiple populations. Practically, this suggests, for example, that while individuals distributed in Seed Zone 1 (see red in Figure 3 from St. Clair et al., 2013) in the western, central, and eastern Central Basin and Range ecoregion may have similar phenotypic characteristics that are putatively adapted to that specific climate, they also have different genetic backgrounds that should be considered prior to transferring plant materials across this ecoregion. The discordance between populations and level III ecoregions exemplifies the practical knowledge gained from investigating geographic patterns of genetic variation and population histories—namely, resolving populations and their histories facilitates the identification of broad‐scale seed transfer zones so that practitioners do not have to rely on environmental proxies (e.g., ecoregions) that likely correspond poorly with the biology and history of a species of interest (e.g., Lesica, Adams, & Smith, 2016).

#### 4.2.2 Genetic diversity and *P. spicata* commercial germplasm sources

Including commercial germplasm sources in our study design allowed us to assess their genetic diversity in relation to each other and wildland *P. spicata*. Genetic diversity within the commercial germplasm sources is, on average, similar to or greater than the diversity represented by the four *P. spicata* populations (Figure 5). However, there are wildland sampling localities in most populations that exceed the levels of diversity represented by the commercial germplasm sources. These results contrast with the genetic diversity patterns resolved for other developed restoration materials (e.g., Broadhurst, Hopley, Li, & Begley, 2017). Furthermore, when expected heterozygosity and nucleotide diversity are directly compared between the commercial germplasm sources and all of the wildland sampling localities surrounding their putative origins (this includes some, but not all, of the sampling localities in the P/W and WGB populations), the median expected heterozygosity and nucleotide diversity are higher for the commercial germplasm sources than the wildland sampling localities (Supporting Information Table S2, graphic representation not shown). We hypothesize that the elevated diversity of commercial germplasm sources results from heterosis. For example, if developers of the commercial germplasm sources continuously selected individuals and/or populations that had elevated performance metrics and these were, in part, due to elevated heterozygosity (Stuber, 1994), this variation should still be present. High genetic diversity is a goal of plant materials development and resolving the processes that have generated/maintained genetic diversity in commercial germplasm sources may be informative for developing future restoration materials.

### 4.3 Conclusion

Investigating a species’ genetic variation can play a foundational role in the use and development of native plant restoration materials. Characterizing the genetic diversity and geographic distribution of populations can guide the development of diverse and representative plant materials. Furthermore, generating estimates of divergence times and migration rates among populations can provide restoration professionals with knowledge to deploy appropriate materials to project sites, with the goal of supporting species and community resilience and improving restoration outcomes.

## DATA ARCHIVING STATEMENT

Data for this study will be available at the Dryad Digital Repository: https://doi.org/10.5061/dryad.rc1jr0v.

## CONFLICT OF INTEREST

None declared.
