# Supplementary material for: Population history provides foundational knowledge for utilizing and developing native plant restoration materials
Source: Evol Appl. 2018 Sep 24;11(10):2025–39. doi: 10.1111/eva.12704 (PMC6231468; doi:10.1111/eva.12704)
Supplement: Supplementary file 1 [file EVA-11-2025-s001.docx]

Figure S1. Distribution of *Pseudoroegneria spicata* individuals (blue) and *Elymus wawawaiensis* individuals (red) along PC1 and PC2 axes of genetic variation. Percent variation explained by each axis is given in parentheses. Individuals from the Secar and Discovery propagated lines (i.e., the known *E. wawawaiensis* individuals) are distributed within the black circle, with one exception.

Figure S2. *Pseudoroegneria spicata* sPCA eigenvalues. Positive eigenvalues denote global structures (a) and are decomposed into their variance and Moran’s *I* spatial components (b). The first three positive eigenvalues explain 49.4% of the genetic variation and are well separated from subsequent eigenvalues, as evidenced by the magnitude of difference between the red bars and grey bars in (a) and the separation of the circled λ values in (b). This pattern supports interpreting the first three global structures.

Figure S3. Dominant axes of genetic variation resolved using sPCA. In each plot, the gradient from yellow to blue represents genetic similarity across the landscape. This information is reiterated by the black and white squares, which represent individuals and that are jittered around their sampling locations. Big black squares are most genetically dissimilar from big white squares.

Latitude

Longitude

Figure S4. Individuals colored by genetic similarity when accounting for the three dominant global sPCA structures (sPC1, sPC2, and sPC3). Individuals are graphed in geographic space according to their sampling localities, and they are jittered so that they do not completely overlap. More similar colors represent more similar genetic identities.

Figure S5. Maximum likelihood tree calculated with PhyML (Guindon et al. 2010; Lefort et al. 2017). Branch support is measured from 1000 bootstrap replicates. See Figure 4 and Table S1 for population descriptions.

Table S1. Details of sampling localities, data processing, and analyses. Columns indicate: total number of individuals sampled per locality (# Ind); number of individuals per locality used in structure, sPCA, and for calculation of diversity statistics (# Ind analyzed); sampling locality coordinates (Latitude/Longitude); ploidy level of sampling locality (M denotes multiple ploidy levels were recovered across individuals); average # reads per individual per sampling locality after data processing (Processed reads); average # reads per individual per sampling locality used to construct GBS loci (Utilized reads); population that a sampling locality was included in for demographic modeling with fastsimcoal2 (FSC2 population). Bolded ploidy values indicate that the tetraploid individuals from the sampling locality cluster with known *Elymus wawawaiensis* individuals from the Discovery and Secar propagated lines in PC space.

| Sampling locality | # Ind | # Ind analyzed (post PCA) | Latitude | Longitude | Ploidy | Processed reads per individual ± SD | Utilized reads per individual ± SD | FSC2 population |
| --- | --- | --- | --- | --- | --- | --- | --- | --- |
| BBWG001 | 4 | 0 | 40.30701 | -116.89968 | 2x | 2856175 ±70964.4 | 1436482 ±102475.2 | - |
| BBWG002 | 5 | 5 | 41.61463 | -117.54055 | 2x | 2118842 ±342277.6 | 703973.6 ±96800.98 | - |
| BBWG004 | 5 | 0 | 41.21215 | -116.53765 | M | 2537381 ±476197.2 | 1217399 ±208376.1 | - |
| BBWG005 | 4 | 4 | 41.61463 | -117.54055 | 2x | 3202630 ±877662 | 1441672 ±483807.5 | - |
| BBWG006 | 5 | 5 | 41.53608 | -117.72983 | 2x | 1977929 ±159955.1 | 881162 ±75051.58 | - |
| BBWG007 | 5 | 5 | 40.92103 | -117.65048 | 2x | 2763694 ±769650.8 | 965349.6 ±304389.9 | - |
| BBWG008 | 5 | 5 | 41.20113 | -116.38633 | 2x | 2423615 ±243779 | 1135205 ±106650.9 | - |
| BBWG010 | 4 | 3 | 41.2111 | -116.32253 | 2x | 2777404 ±320981 | 1304781 ±146662.3 | - |
| BBWG011 | 5 | 5 | 40.92103 | -117.65048 | 2x | 3307521 ±431965.1 | 582262.6 ±105576.3 | - |
| BBWG012 | 5 | 5 | 41.5755 | -115.95311 | 2x | 2092156 ±98546.19 | 929162.8 ±134177.1 | - |
| BBWG014 | 4 | 4 | 40.86448 | -115.25233 | 2x | 2917213 ±216038.1 | 934410.5 ±84222.11 | - |
| BBWG017 | 4 | 4 | 41.25321 | -116.06843 | 2x | 2761758 ±677389.8 | 1342717 ±392143.4 | - |
| BBWG018 | 4 | 4 | 41.11633 | -114.79495 | 2x | 2371545 ±1289485 | 1011003 ±531070.8 | - |
| BBWG019 | 5 | 5 | 41.54106 | -115.74993 | 2x | 2392721 ±609283.4 | 465867.2 ±98416.9 | - |
| BBWG020 | 5 | 5 | 41.54106 | -115.74993 | 2x | 2794708 ±389712.6 | 1324062 ±184291.1 | - |
| BBWG024 | 5 | 5 | 41.5201 | -111.74891 | 2x | 2647262 ±451063.1 | 458491 ±89286.08 | - |
| BBWG026 | 4 | 4 | 41.59145 | -111.74153 | 2x | 2748783 ±382355.1 | 944702.8 ±158170.5 | - |

Table S1. Continued

| Sampling locality | # Ind | # Ind analyzed (post PCA) | Latitude | Longitude | Ploidy | Processed reads per individual ± SD | Utilized reads per individual ± SD | FSC2 population |
| --- | --- | --- | --- | --- | --- | --- | --- | --- |
| BBWG030 | 4 | 4 | 41.83026 | -111.77686 | 2x | 3327526 ±495084.6 | 541955.5 ±57788.3 | - |
| BBWG031 | 5 | 5 | 41.5961 | -111.78465 | 2x | 2606313 ±857310.3 | 436522 ±142877.8 | - |
| BBWG033 | 5 | 5 | 41.608 | -111.97225 | 2x | 3381720 ±827468.5 | 551524.2 ±111451.3 | - |
| BBWG040 | 5 | 5 | 40.86694 | -115.86912 | 2x | 2191745 ±265920.9 | 1057574 ±163915.7 | - |
| BBWG041 | 4 | 4 | 41.52047 | -116.50006 | 2x | 1626389 ±294962.1 | 792786.2 ±163835.4 | - |
| BBWG042 | 5 | 5 | 41.66178 | -112.05248 | 2x | 2575333 ±450445 | 1210912 ±185420.7 | - |
| BBWG043 | 4 | 4 | 41.95777 | -114.8095 | 2x | 2497992 ±324090 | 1033145 ±203569.1 | - |
| BBWG044 | 5 | 5 | 38.62466 | -112.81927 | 2x | 4175731 ±3124346 | 1846740 ±1073926 | - |
| BBWG045 | 4 | 4 | 41.21335 | -117.71264 | 2x | 3787753 ±1408102 | 1309347 ±519006 | - |
| BBWG046 | 4 | 4 | 39.86176 | -111.5439 | 2x | 2914648 ±862129.5 | 1233011 ±394836.8 | WAS |
| BBWG047 | 5 | 5 | 41.58548 | -111.56325 | 2x | 3001501 ±504856.5 | 1480555 ±274663.7 | - |
| BBWG048 | 4 | 4 | 40.64525 | -111.8027 | 2x | 1858991 ±91612.13 | 753726.8 ±60411.18 | WAS |
| BBWG050 | 4 | 4 | 39.95352 | -111.77837 | 2x | 4208862 ±2986708 | 1353842 ±944322.4 | - |
| BBWG051 | 5 | 5 | 41.24742 | -113.99326 | 2x | 3141613 ±692588.6 | 1374317 ±270271.6 | - |
| BBWG052 | 4 | 4 | 40.7457 | -116.8686 | 2x | 2026206 ±182924.5 | 880609.2 ±93599.22 | - |
| BBWG053 | 4 | 4 | 41.38805 | -112.026 | 2x | 3779763 ±476175.2 | 1650498 ±231253.6 | - |
| BBWG054 | 5 | 5 | 40.16137 | -111.50765 | 2x | 1995761 ±389392.1 | 907376.8 ±157248.2 | WAS |
| BBWG055 | 5 | 5 | 41.52643 | -113.66996 | 2x | 1994182 ±287941.7 | 888210.6 ±163363.6 | - |
| BBWG056 | 5 | 5 | 39.43825 | -111.53358 | 2x | 2653859 ±321261.2 | 893805.2 ±110982.2 | - |
| BBWG057 | 5 | 5 | 39.56294 | -115.78257 | 2x | 2691233 ±746733 | 1265812 ±382994.2 | - |
| BBWG058 | 5 | 5 | 39.27252 | -111.6273 | 2x | 3211883 ±868259.3 | 535064.4 ±128826.3 | - |
| BBWG059 | 4 | 4 | 39.8332 | -112.24623 | 2x | 4673843 ±2376377 | 1410929 ±1051563 | - |
| BBWG060 | 5 | 4 | 40.87847 | -114.26487 | 2x | 2234936 ±135928.2 | 946774 ±65874.69 | - |
| BBWG063 | 5 | 5 | 39.89483 | -111.88918 | 2x | 2642766 ±148389.2 | 900673.2 ±68956.27 | - |

Table S1. Continued.

| Sampling locality | # Ind | # Ind analyzed (post PCA) | Latitude | Longitude | Ploidy | Processed reads per individual ± SD | Utilized reads per individual ± SD | FSC2 population |
| --- | --- | --- | --- | --- | --- | --- | --- | --- |
| BBWG064 | 4 | 4 | 40.20469 | -113.9041 | 2x | 2090309 ±78741.45 | 916032.8 ±33663.59 | - |
| BBWG065 | 4 | 4 | 40.9412 | -109.71353 | 2x | 1842803 ±440039.1 | 824807.5 ±181219.3 | - |
| BBWG068 | 5 | 5 | 40.6527 | -111.806 | 2x | 2099570 ±279493.4 | 957550 ±149356.2 | - |
| BBWG069 | 5 | 5 | 39.98225 | -112.22479 | 2x | 1923818 ±210820.7 | 838355 ±79343.55 | WAS |
| BBWG070 | 4 | 4 | 40.00396 | -111.73251 | 2x | 2053773 ±115805.1 | 906239 ±52717.9 | WAS |
| BBWG072 | 5 | 5 | 39.76511 | -112.11444 | 2x | 2765998 ±426733.9 | 907267 ±155632.9 | - |
| BBWG073 | 5 | 5 | 40.48312 | -110.50974 | 2x | 3172291 ±800494.6 | 1313885 ±258844.4 | - |
| BBWG074 | 5 | 5 | 39.47039 | -111.88813 | 2x | 2197569 ±305136.3 | 884303 ±160201.6 | WAS |
| BBWG075 | 5 | 5 | 39.0793 | -111.70349 | 2x | 2053311 ±359668.4 | 926287.8 ±186773.4 | WAS |
| BBWG076 | 5 | 4 | 38.592077 | -114.71975 | 2x | 1806747 ±287742.5 | 714086.4 ±176516.5 | - |
| BBWG077 | 5 | 5 | 41.06384 | -111.69986 | 2x | 2274584 ±635937.5 | 1026475 ±383842.4 | - |
| BBWG078 | 5 | 5 | 39.48644 | -111.99565 | 2x | 2727468 ±655441.1 | 1168766 ±310164.5 | WAS |
| BBWG080 | 5 | 4 | 39.08826 | -112.22057 | 2x | 2418631 ±521506.6 | 1114851 ±287151.5 | WAS |
| BBWG081 | 5 | 4 | 40.42336 | -109.78308 | 2x | 2637676 ±669632.6 | 1317501 ±381092.4 | - |
| BBWG082 | 5 | 5 | 40.41329 | -111.74454 | 2x | 2324564 ±275537.6 | 1080206 ±117699.4 | WAS |
| BBWG084 | 4 | 4 | 39.45077 | -115.93431 | 2x | 2806018 ±93132.81 | 1363722 ±40628.38 | - |
| BBWG085 | 5 | 4 | 41.49206 | -111.95187 | 2x | 2331789 ±294607.8 | 991305.2 ±186423.1 | - |
| BBWG087 | 5 | 5 | 40.78668 | -111.41779 | 2x | 1895440 ±338346 | 877783.4 ±155673.5 | WAS |
| BBWG088 | 5 | 5 | 41.05631 | -114.32889 | 2x | 2349874 ±560216.1 | 1073538 ±246890.8 | - |
| BBWG089 | 5 | 5 | 41.66103 | -114.98103 | 2x | 2878253 ±212931.8 | 1462376 ±114169.5 | - |
| BBWG093 | 5 | 5 | 41.31093 | -117.70506 | 2x | 2967972 ±435089.3 | 510174.2 ±88566.98 | - |
| BBWG094 | 5 | 5 | 41.62517 | -111.7968 | 2x | 2216523 ±161577.8 | 995369.6 ±102360.9 | - |
| BBWG096 | 5 | 5 | 39.80557 | -111.83493 | 2x | 2454307 ±307513.1 | 1149681 ±162336.7 | WAS |
| BBWG097 | 4 | 4 | 40.29771 | -115.55431 | 2x | 2386276 ±241711.8 | 1152105 ±130481 | - |
| BBWG099 | 5 | 5 | 39.26671 | -114.95135 | 2x | 2729511 ±272824.9 | 1244485 ±246081.7 | - |

Table S1. Continued.

| Sampling locality | # Ind | # Ind analyzed (post PCA) | Latitude | Longitude | Ploidy | Processed reads per individual ± SD | Utilized reads per individual ± SD | FSC2 population |
| --- | --- | --- | --- | --- | --- | --- | --- | --- |
| BBWG100 | 5 | 5 | 39.59142 | -111.82328 | 2x | 3373286 ±1035075 | 1485239 ±463948.6 | WAS |
| BBWG101 | 4 | 4 | 41.98554 | -111.42614 | 2x | 2450400 ±80581.63 | 1163951 ±86404.24 | - |
| BBWG102 | 5 | 5 | 39.34408 | -111.5669 | 2x | 1828162 ±326910.3 | 807744.8 ±144814.2 | WAS |
| BBWG252 | 5 | 0 | - | - | 2x | 3018569 ±410858.3 | 525535 ±62866.49 | - |
| BBWG253 | 5 | 0 | - | - | 4x | 2426910 ±468690.1 | 835471.8 ±150848.5 | - |
| BBWG254 | 5 | 5 | 43.58344 | -113.10821 | 2x | 3342376 ±355857.5 | 577307 ±48741.66 | - |
| BBWG256 | 5 | 5 | 43.22252 | -118.46978 | 2x | 3343868 ±801527.2 | 1141597 ±288551.9 | - |
| BBWG258 | 5 | 0 | - | - | **M** | 2709397 ±346740.1 | 807033.2 ±240087.1 | - |
| BBWG259 | 4 | 4 | 43.5866 | -120.0219 | NA | 2334139 ±161178.4 | 839008.5 ±85301.99 | - |
| BBWG260 | 5 | 5 | 43.65899 | -118.60128 | 2x | 2785797 ±485707.6 | 447807.8 ±86875.94 | - |
| BBWG261 | 5 | 5 | 46.966931 | -118.822888 | 2x | 2179237 ±527353.2 | 735030.6 ±157788.6 | - |
| N1-139 | 4 | 4 | 46.3016 | -120.21562 | 2x | 2349312 ±368477.5 | 897425.5 ±316842.1 | WGB |
| N1-149 | 5 | 5 | 47.30084 | -119.53822 | 2x | 2362926 ±476319.8 | 1061576 ±456796.8 | WGB |
| N1-152 | 7 | 7 | 47.22593 | -119.40073 | 2x | 2127040 ±230914.7 | 882830.3 ±199917.3 | WGB |
| N1-156 | 7 | 7 | 47.51479 | -119.50023 | 2x | 2825844 ±251160.2 | 1143299 ±139325.2 | WGB |
| N3A-123 | 7 | 6 | 45.83204 | -120.53102 | 2x | 2525770 ±344038.5 | 1028627 ±170258.2 | WGB |
| N3A-133 | 7 | 7 | 46.15657 | -120.00919 | 2x | 2298666 ±216682.1 | 903997.3 ±222809.7 | WGB |
| N3A-143 | 7 | 7 | 46.64094 | -118.85801 | 2x | 2408898 ±251892.5 | 989621.3 ±201540.6 | P/W |
| N3A-148 | 7 | 7 | 46.97286 | -118.58993 | 2x | 2619464 ±256438.6 | 1122129 ±196626.3 | P/W |
| N3A-160 | 6 | 5 | 47.65635 | -119.65857 | 2x | 2497674 ±345566.2 | 1065265 ±250842.3 | WGB |
| N4-220 | 7 | 7 | 44.43008333 | -117.5223611 | 2x | 2824527 ±579912.7 | 1269173 ±305272.2 | - |
| N4-224 | 7 | 7 | 44.56811111 | -117.5994722 | 2x | 2421281 ±433618.4 | 1039782 ±255181.1 | - |
| N4-226 | 7 | 7 | 44.64444444 | -117.8684444 | 2x | 2756326 ±244429 | 1309469 ±294985.2 | - |
| N4-227 | 7 | 7 | 44.64866667 | -117.4716389 | 2x | 2372481 ±554756.3 | 876002.4 ±24588.1 | - |
| N4-234 | 7 | 7 | 44.8265 | -117.4547778 | 2x | 2505623 ±455792.8 | 1014769 ±310975.9 | - |

Table S1. Continued.

| Sampling locality | # Ind | # Ind analyzed (post PCA) | Latitude | Longitude | Ploidy | Processed reads per individual ± SD | Utilized reads per individual ± SD | FSC2 population |
| --- | --- | --- | --- | --- | --- | --- | --- | --- |
| N5-111 | 7 | 0 | 45.33004 | -117.20723 | **M** | 2523763 ±526965.3 | 953866.6 ±52606.49 | - |
| N5-116 | 7 | 0 | 45.54268 | -117.43397 | **4x** | 2412703 ±476205 | 927971.3 ±123701.5 | - |
| N5-120 | 7 | 7 | 45.628 | -117.59927 | 2x | 2683155 ±189519.6 | 1119358 ±225935.4 | P/W |
| N5-122 | 7 | 7 | 45.80508 | -117.38867 | 2x | 2063614 ±177573.6 | 807089.3 ±221338.5 | P/W |
| N5-243 | 7 | 0 | 45.40503 | -117.22891 | **M** | 2304335 ±437417 | 801339.7 ±164802.4 | - |
| N6A-115 | 7 | 6 | 45.46726 | -119.00622 | 2x | 2362488 ±258364.9 | 966372.3 ±186634.6 | P/W |
| N6A-124 | 4 | 0 | 45.90529 | -118.92276 | **4x** | 2496887 ±202221.7 | 864307.8 ±133043.8 | - |
| N6A-134 | 7 | 7 | 46.19327 | -118.11021 | 2x | 2314319 ±238280.3 | 925737.3 ±180800.3 | P/W |
| N6A-141 | 7 | 7 | 46.43291 | -117.93803 | 2x | 2530197 ±418493.6 | 977237.6 ±128274.3 | P/W |
| N6A-144 | 7 | 7 | 46.6419 | -117.76384 | 2x | 2346538 ±379900.5 | 896062.3 ±150394.2 | P/W |
| N6B-118 | 7 | 7 | 45.6013 | -118.6133 | 2x | 2359238 ±468616.7 | 895197 ±99938.19 | P/W |
| N6B-130 | 7 | 7 | 46.06256 | -117.23254 | 2x | 2163801 ±431917.5 | 807177.3 ±111898.4 | P/W |
| N6B-137 | 7 | 7 | 46.26236 | -117.41138 | 2x | 1980290 ±271974.7 | 742892.4 ±123481 | P/W |
| N6B-140 | 7 | 7 | 46.3162 | -117.57042 | 2x | 2233561 ±582869.8 | 845402.9 ±162716.5 | P/W |
| N6B-147 | 7 | 6 | 46.83891 | -117.28145 | 2x | 2119906 ±452815.4 | 773444.7 ±216257.5 | P/W |
| N7A-103 | 7 | 7 | 44.83662 | -119.02749 | 2x | 1919088 ±422129.1 | 727311 ±92801.1 | WGB |
| N7A-105 | 7 | 5 | 44.90707 | -120.276 | 2x | 1889177 ±448001.9 | 685125 ±120015.4 | WGB |
| N7A-229 | 7 | 7 | 44.7275 | -119.9597222 | 2x | 2450781 ±452489.6 | 1000513 ±295192 | WGB |
| N7A-236 | 7 | 7 | 44.94555556 | -120.7213889 | 2x | 2582379 ±289341 | 1274506 ±138812.6 | WGB |
| N7A-237 | 7 | 7 | 44.94666667 | -120.7875 | 2x | 2522165 ±421229.9 | 1304050 ±260217.3 | - |
| N7B-114 | 7 | 7 | 45.46434 | -118.19721 | 2x | 2014689 ±399131.2 | 895463.3 ±252481.5 | P/W |
| N7B-119 | 7 | 7 | 45.62631 | -118.13402 | 2x | 2194281 ±710844.7 | 1022289 ±397258 | P/W |
| N7B-126 | 7 | 7 | 45.96175 | -118.02188 | 2x | 2082743 ±459773.1 | 977760.1 ±290797.9 | P/W |
| N7B-132 | 7 | 7 | 46.13915 | -117.76534 | 2x | 2210584 ±406129.5 | 1043145 ±250718.2 | P/W |
| N7B-136 | 7 | 7 | 46.22359 | -117.77087 | 2x | 2098149 ±568705.3 | 964942.4 ±312194.3 | P/W |

Table S1. Continued.

| Sampling locality | # Ind | # Ind analyzed (post PCA) | Latitude | Longitude | Ploidy | Processed reads per individual ± SD | Utilized reads per individual ± SD | FSC2 population |
| --- | --- | --- | --- | --- | --- | --- | --- | --- |
| S1-194 | 7 | 5 | 43.30136111 | -113.7925556 | 2x | 2931018 ±259241.4 | 1408219 ±271752.3 | EGB |
| S1-201 | 7 | 5 | 43.06116667 | -114.8259167 | 2x | 3136146 ±362802.5 | 1445255 ±225042.5 | - |
| S1-207 | 7 | 7 | 43.07588889 | -114.8609722 | 2x | 2769964 ±259389.3 | 1280962 ±225014.3 | - |
| S2A-196 | 7 | 6 | 43.33913889 | -112.9513056 | 2x | 3594491 ±468997.4 | 1954497 ±327304.3 | EGB |
| S2A-198 | 6 | 6 | 43.37744444 | -113.0376111 | 2x | 2903049 ±571291.3 | 1574397 ±348264.7 | EGB |
| S2A-200 | 7 | 7 | 43.43716667 | -113.1134167 | 2x | 3591337 ±621929.8 | 1782694 ±312735.8 | EGB |
| S2A-202 | 7 | 7 | 43.54133333 | -112.6380278 | 2x | 2544283 ±723233.9 | 1324980 ±436251.8 | EGB |
| S2A-205 | 7 | 7 | 43.58094444 | -113.1136667 | 2x | 3506200 ±595775.6 | 1747848 ±298598.9 | EGB |
| S3A-208 | 7 | 0 | 43.73491667 | -117.2780556 | **4x** | 2687666 ±372151.6 | 1260432 ±216904.8 | - |
| S3A-209 | 7 | 6 | 43.76447222 | -117.7197778 | 2x | 2531092 ±536990 | 1184818 ±279675.8 | - |
| S3A-214 | 7 | 0 | 44.05330556 | -117.1683611 | **4x** | 2878609 ±646803.8 | 1328136 ±266912.2 | - |
| S3A-215 | 7 | 0 | 44.08688889 | -117.8210833 | **M** | 3218305 ±611055.6 | 1567269 ±298982.6 | - |
| S4-176 | 6 | 6 | 42.82844444 | -117.1542222 | 2x | 3264556 ±301585 | 1636666 ±306706.8 | - |
| S4-180 | 7 | 7 | 42.88169444 | -117.3886667 | 2x | 3064553 ±401660.9 | 1588577 ±247883.4 | - |
| S4-193 | 7 | 7 | 43.26772222 | -117.0054722 | 2x | 2963492 ±330427.6 | 1527791 ±260007.1 | - |
| S4-197 | 7 | 7 | 43.36647222 | -117.1193333 | 2x | 3263776 ±378344.1 | 1647760 ±193287.4 | - |
| S4-212 | 7 | 7 | 43.14602778 | -117.4619167 | 2x | 3906671 ±717587.7 | 2007135 ±386503.3 | - |
| S5-187 | 7 | 7 | 43.02277778 | -116.8178333 | 2x | 2704488 ±377255.1 | 1389790 ±219284.2 | - |
| S5-192 | 7 | 7 | 43.24538889 | -116.8525278 | 2x | 2615862 ±501346.3 | 1360618 ±295860.7 | - |
| S5-195 | 7 | 7 | 43.04741667 | -116.8787778 | 2x | 3001342 ±366955.9 | 1518287 ±151039.8 | - |
| S5-211 | 7 | 7 | 43.11922222 | -116.73125 | 2x | 3308024 ±326739.9 | 1758727 ±173716.1 | - |
| S5-240 | 7 | 7 | 42.99102778 | -116.8973056 | 2x | 2769505 ±358103.6 | 1284873 ±246262.4 | - |
| S6A-161 | 7 | 7 | 41.64730556 | -117.2674444 | 2x | 2420322 ±643639.8 | 1195917 ±436463.9 | - |
| S6A-163 | 7 | 7 | 41.70244444 | -117.0307778 | 2x | 2299162 ±1184769 | 1176694 ±734708.1 | - |
| S6A-171 | 7 | 7 | 41.9885 | -117.4706667 | 2x | 2159600 ±404073.7 | 1046112 ±288115.2 | - |

Table S1. Continued.

| Sampling locality | # Ind | # Ind analyzed (post PCA) | Latitude | Longitude | Ploidy | Processed reads per individual ± SD | Utilized reads per individual ± SD | FSC2 population |
| --- | --- | --- | --- | --- | --- | --- | --- | --- |
| S6A-172 | 7 | 7 | 41.99288889 | -117.5301944 | 2x | 1789896 ±491970.5 | 730282.3 ±181529.5 | - |
| S6A-191 | 7 | 0 | - | - | **M** | 2540453 ±351954.7 | 1134621 ±255318.4 | - |
| S6A-242 | 7 | 7 | 41.78844444 | -117.0424722 | 2x | 2280929 ±189261.3 | 1011936 ±152561.9 | - |
| S6B-162 | 7 | 7 | 41.69358333 | -117.3272222 | 2x | 1941146 ±457275.9 | 826634.9 ±177862 | - |
| S6B-167 | 7 | 7 | 41.79163889 | -117.2975 | 2x | 1972028 ±497865.6 | 843935.3 ±190977.1 | - |
| S6B-168 | 7 | 7 | 41.81855556 | -117.3667778 | 2x | 1978979 ±427315.5 | 837071.9 ±179578.4 | - |
| S6B-169 | 7 | 7 | 41.95516667 | -117.4045 | 2x | 1872147 ±689024.8 | 772362.7 ±265657.5 | - |
| S6B-241 | 7 | 7 | 41.79161111 | -117.2265833 | 2x | 2313802 ±184641.5 | 1024019 ±61746.33 | - |
| S7B-173 | 7 | 7 | 42.45 | -118.7483333 | 2x | 1885482 ±572343.8 | 786188.7 ±207235.4 | - |
| S7B-174 | 11 | 7 | 42.64944444 | -118.7419444 | 2x | 1803303 ±616186.7 | 801267.7 ±324068.2 | - |
| S7B-175 | 7 | 7 | 42.76166667 | -118.7266667 | 2x | 1821375 ±850865 | 849981 ±445557.2 | - |
| S7B-183 | 7 | 7 | 42.96052778 | -118.5394167 | 2x | 2582007 ±278009.3 | 1238321 ±179353 | - |
| S7B-184 | 6 | 3 | 42.97252778 | -118.5362222 | 2x | 1368188 ±1401853 | 643484 ±654542.4 | - |
| Anatone | 10 | 10 | - | - | 2x | 2734549 ±390967.1 | 1071907 ±188204.6 | - |
| Columbia | 10 | 10 | - | - | 2x | 2652960 ±489326.7 | 1146801 ±212459.3 | - |
| Discovery | 10 | 0 | - | - | **4x** | 2485514 ±185005.5 | 1069838 ±84159.52 | - |
| Goldar | 10 | 10 | - | - | 2x | 2765237 ±527351.4 | 1074038 ±233689.2 | - |
| P-7 | 10 | 9 | - | - | **M** | 2453891 ±226819.7 | 1014858 ±107943 | - |
| Secar | 10 | 0 | - | - | **4x** | 2417788 ±404298.8 | 1000364 ±199512.2 | - |
| Wahluke | 10 | 10 | - | - | 2x | 2504142 ±367139.5 | 1096848 ±188344.7 | - |
| Whitmar | 10 | 10 | - | - | 2x | 2366698 ±320952.7 | 933052.8 ±135174.2 | - |

| Population | Variant Sites | Private | *P* | *H_OBS_* | *H_EXP_* | π | *F_IS_* |
| --- | --- | --- | --- | --- | --- | --- | --- |
| BBWG002 | 163176 | 46 | 0.980 | 0.019 | 0.025 | 0.030 | 0.020 |
| BBWG005 | 163115 | 74 | 0.978 | 0.025 | 0.027 | 0.033 | 0.014 |
| BBWG006 | 141236 | 70 | 0.973 | 0.021 | 0.034 | 0.040 | 0.036 |
| BBWG007 | 136661 | 42 | 0.977 | 0.020 | 0.029 | 0.034 | 0.027 |
| BBWG008 | 158495 | 295 | 0.970 | 0.025 | 0.039 | 0.047 | 0.039 |
| BBWG010 | 140609 | 145 | 0.972 | 0.027 | 0.035 | 0.043 | 0.028 |
| BBWG011 | 164279 | 19 | 0.980 | 0.016 | 0.025 | 0.030 | 0.026 |
| BBWG012 | 143975 | 82 | 0.976 | 0.020 | 0.031 | 0.037 | 0.033 |
| BBWG014 | 135048 | 65 | 0.979 | 0.021 | 0.027 | 0.032 | 0.021 |
| BBWG017 | 148641 | 187 | 0.972 | 0.027 | 0.036 | 0.044 | 0.030 |
| BBWG018 | 148463 | 113 | 0.980 | 0.014 | 0.025 | 0.030 | 0.028 |
| BBWG019 | 152353 | 28 | 0.979 | 0.014 | 0.026 | 0.032 | 0.032 |
| BBWG020 | 143927 | 130 | 0.972 | 0.024 | 0.037 | 0.044 | 0.036 |
| BBWG024 | 135842 | 25 | 0.986 | 0.012 | 0.018 | 0.021 | 0.017 |
| BBWG026 | 120859 | 53 | 0.984 | 0.017 | 0.020 | 0.024 | 0.012 |
| BBWG030 | 140801 | 19 | 0.983 | 0.014 | 0.021 | 0.026 | 0.021 |
| BBWG031 | 152772 | 30 | 0.985 | 0.011 | 0.019 | 0.023 | 0.020 |
| BBWG033 | 148417 | 27 | 0.980 | 0.015 | 0.025 | 0.031 | 0.028 |
| BBWG040 | 147213 | 123 | 0.975 | 0.022 | 0.032 | 0.038 | 0.029 |
| BBWG041 | 144889 | 25 | 0.977 | 0.016 | 0.028 | 0.035 | 0.034 |
| BBWG042 | 152640 | 185 | 0.979 | 0.019 | 0.027 | 0.033 | 0.026 |
| BBWG043 | 133571 | 76 | 0.976 | 0.019 | 0.030 | 0.036 | 0.030 |
| BBWG044 | 139110 | 100 | 0.978 | 0.020 | 0.029 | 0.034 | 0.026 |
| BBWG045 | 152313 | 59 | 0.975 | 0.023 | 0.032 | 0.039 | 0.028 |
| BBWG046 | 158656 | 84 | 0.976 | 0.023 | 0.030 | 0.036 | 0.023 |
| BBWG047 | 152026 | 159 | 0.974 | 0.021 | 0.034 | 0.041 | 0.039 |
| BBWG048 | 144361 | 38 | 0.979 | 0.018 | 0.027 | 0.033 | 0.026 |
| BBWG050 | 151069 | 33 | 0.980 | 0.018 | 0.026 | 0.031 | 0.023 |
| BBWG051 | 134008 | 310 | 0.977 | 0.025 | 0.031 | 0.036 | 0.022 |
| BBWG052 | 140706 | 249 | 0.975 | 0.020 | 0.032 | 0.039 | 0.033 |

Table S2. Genetic summary statistics for the diploid *P. spicata* sampling localities and propagated lines, as calculated by the populations program in stacks. Results are presented for variant nucleotide positions only. Shown are: the total number of variant sites; the total number of private alleles (Private); the average frequency of the major allele (*P*), the average observed heterozygosity (*H_OBS_*), the average expected heterozygosity (*H_EXP_*), the average nucleotide diversity (π), and the average Wright’s inbreeding coefficient (*F_IS_*).

Table S2. Continued

| Population | Variant Sites | Private | *P* | *H_OBS_* | *H_EXP_* | π | *F_IS_* |
| --- | --- | --- | --- | --- | --- | --- | --- |
| BBWG054 | 123588 | 56 | 0.979 | 0.018 | 0.027 | 0.032 | 0.027 |
| BBWG055 | 151077 | 182 | 0.980 | 0.017 | 0.026 | 0.031 | 0.025 |
| BBWG056 | 125356 | 61 | 0.973 | 0.019 | 0.034 | 0.041 | 0.042 |
| BBWG057 | 147761 | 411 | 0.971 | 0.026 | 0.037 | 0.044 | 0.035 |
| BBWG058 | 142868 | 23 | 0.981 | 0.014 | 0.024 | 0.029 | 0.028 |
| BBWG059 | 118472 | 48 | 0.977 | 0.022 | 0.028 | 0.035 | 0.022 |
| BBWG063 | 183073 | 59 | 0.979 | 0.018 | 0.027 | 0.032 | 0.026 |
| BBWG064 | 178209 | 149 | 0.981 | 0.016 | 0.024 | 0.030 | 0.024 |
| BBWG065 | 88997 | 191 | 0.981 | 0.015 | 0.024 | 0.029 | 0.026 |
| BBWG068 | 168759 | 56 | 0.978 | 0.020 | 0.028 | 0.033 | 0.025 |
| BBWG069 | 160488 | 50 | 0.977 | 0.016 | 0.029 | 0.035 | 0.036 |
| BBWG070 | 173555 | 37 | 0.979 | 0.018 | 0.027 | 0.033 | 0.025 |
| BBWG072 | 182311 | 64 | 0.978 | 0.018 | 0.028 | 0.034 | 0.028 |
| BBWG074 | 176272 | 70 | 0.977 | 0.019 | 0.030 | 0.035 | 0.030 |
| BBWG075 | 187863 | 58 | 0.977 | 0.020 | 0.030 | 0.036 | 0.029 |
| BBWG076 | 176895 | 133 | 0.972 | 0.019 | 0.035 | 0.042 | 0.042 |
| BBWG077 | 177118 | 88 | 0.976 | 0.020 | 0.030 | 0.036 | 0.029 |
| BBWG080 | 181067 | 365 | 0.965 | 0.033 | 0.045 | 0.053 | 0.037 |
| BBWG081 | 184713 | 472 | 0.964 | 0.034 | 0.047 | 0.056 | 0.040 |
| BBWG082 | 169058 | 84 | 0.977 | 0.021 | 0.030 | 0.036 | 0.027 |
| BBWG084 | 180594 | 275 | 0.972 | 0.026 | 0.036 | 0.043 | 0.031 |
| BBWG085 | 167604 | 235 | 0.963 | 0.030 | 0.048 | 0.057 | 0.050 |
| BBWG087 | 176346 | 43 | 0.980 | 0.018 | 0.025 | 0.030 | 0.022 |
| BBWG088 | 169672 | 331 | 0.978 | 0.018 | 0.028 | 0.034 | 0.029 |
| BBWG089 | 184067 | 155 | 0.976 | 0.023 | 0.031 | 0.037 | 0.025 |
| BBWG093 | 169026 | 14 | 0.979 | 0.017 | 0.027 | 0.033 | 0.028 |
| BBWG094 | 153128 | 94 | 0.982 | 0.017 | 0.022 | 0.027 | 0.018 |
| BBWG096 | 158041 | 69 | 0.977 | 0.022 | 0.029 | 0.035 | 0.023 |
| BBWG097 | 151151 | 70 | 0.975 | 0.024 | 0.031 | 0.038 | 0.025 |
| BBWG099 | 152205 | 421 | 0.970 | 0.023 | 0.039 | 0.046 | 0.044 |
| BBWG100 | 154313 | 99 | 0.976 | 0.022 | 0.031 | 0.036 | 0.026 |
| BBWG101 | 169543 | 97 | 0.979 | 0.021 | 0.027 | 0.032 | 0.019 |
| BBWG102 | 165676 | 48 | 0.979 | 0.019 | 0.027 | 0.032 | 0.025 |

Table S2. Continued

| Population | Variant Sites | Private | *P* | *H_OBS_* | *H_EXP_* | π | *F_IS_* |
| --- | --- | --- | --- | --- | --- | --- | --- |
| BBWG254 | 99405 | 71 | 0.978 | 0.017 | 0.028 | 0.033 | 0.029 |
| BBWG259 | 117683 | 97 | 0.985 | 0.017 | 0.019 | 0.023 | 0.010 |
| BBWG260 | 116212 | 46 | 0.983 | 0.013 | 0.022 | 0.026 | 0.024 |
| BBWG261 | 73325 | 80 | 0.980 | 0.018 | 0.026 | 0.031 | 0.024 |
| N1_139 | 58326 | 85 | 0.983 | 0.015 | 0.021 | 0.026 | 0.018 |
| N1_149 | 78420 | 100 | 0.981 | 0.016 | 0.025 | 0.030 | 0.025 |
| N1_152 | 110801 | 109 | 0.980 | 0.017 | 0.026 | 0.031 | 0.027 |
| N1_156 | 130169 | 153 | 0.978 | 0.019 | 0.029 | 0.034 | 0.028 |
| N3A_123 | 71000 | 578 | 0.967 | 0.030 | 0.043 | 0.052 | 0.041 |
| N3A_133 | 112833 | 151 | 0.980 | 0.018 | 0.025 | 0.031 | 0.023 |
| N3A_143 | 112027 | 100 | 0.976 | 0.020 | 0.031 | 0.038 | 0.031 |
| N3A_148 | 35600 | 148 | 0.974 | 0.022 | 0.033 | 0.040 | 0.033 |
| N3A_160 | 78908 | 515 | 0.967 | 0.028 | 0.042 | 0.051 | 0.042 |
| N4_220 | 115454 | 406 | 0.964 | 0.030 | 0.046 | 0.056 | 0.046 |
| N4_224 | 121401 | 164 | 0.976 | 0.020 | 0.031 | 0.037 | 0.032 |
| N4_226 | 122874 | 167 | 0.976 | 0.021 | 0.031 | 0.037 | 0.030 |
| N4_227 | 119970 | 116 | 0.977 | 0.020 | 0.029 | 0.036 | 0.028 |
| N4_234 | 141421 | 183 | 0.979 | 0.020 | 0.027 | 0.032 | 0.023 |
| N5_120 | 165947 | 683 | 0.977 | 0.023 | 0.030 | 0.036 | 0.023 |
| N5_122 | 149226 | 160 | 0.980 | 0.016 | 0.026 | 0.031 | 0.027 |
| N6A_115 | 131406 | 527 | 0.960 | 0.035 | 0.052 | 0.063 | 0.051 |
| N6A_134 | 140772 | 220 | 0.974 | 0.022 | 0.033 | 0.040 | 0.033 |
| N6A_141 | 152300 | 157 | 0.976 | 0.021 | 0.031 | 0.037 | 0.030 |
| N6A_144 | 165373 | 123 | 0.976 | 0.020 | 0.030 | 0.037 | 0.030 |
| N6B_118 | 168400 | 307 | 0.978 | 0.018 | 0.028 | 0.034 | 0.028 |
| N6B_130 | 125409 | 77 | 0.980 | 0.018 | 0.026 | 0.032 | 0.025 |
| N6B_137 | 118295 | 95 | 0.980 | 0.015 | 0.025 | 0.030 | 0.027 |
| N6B_140 | 127050 | 120 | 0.977 | 0.017 | 0.030 | 0.036 | 0.034 |
| N6B_147 | 121736 | 346 | 0.967 | 0.027 | 0.043 | 0.052 | 0.045 |
| N7A_229 | 149442 | 337 | 0.975 | 0.020 | 0.031 | 0.038 | 0.031 |
| N7A_236 | 151238 | 387 | 0.973 | 0.026 | 0.034 | 0.041 | 0.028 |
| N7A_237 | 130427 | 368 | 0.974 | 0.023 | 0.033 | 0.040 | 0.030 |
| N7B_119 | 98773 | 348 | 0.978 | 0.017 | 0.027 | 0.033 | 0.030 |
| N7B_126 | 123881 | 235 | 0.978 | 0.019 | 0.028 | 0.034 | 0.026 |
| N7B_132 | 91817 | 131 | 0.978 | 0.019 | 0.028 | 0.034 | 0.027 |
| N7B_136 | 119996 | 129 | 0.979 | 0.018 | 0.027 | 0.032 | 0.026 |
| S1_194 | 175610 | 345 | 0.962 | 0.037 | 0.050 | 0.059 | 0.041 |
| S1_201 | 162359 | 633 | 0.963 | 0.036 | 0.048 | 0.057 | 0.039 |

Table S2. Continued

| Population | Variant Sites | Private | *P* | *H_OBS_* | *H_EXP_* | π | *F_IS_* |
| --- | --- | --- | --- | --- | --- | --- | --- |
| S1_207 | 88536 | 344 | 0.974 | 0.024 | 0.034 | 0.040 | 0.030 |
| S2A_196 | 85409 | 326 | 0.960 | 0.037 | 0.052 | 0.062 | 0.046 |
| S2A_198 | 111626 | 268 | 0.969 | 0.029 | 0.040 | 0.048 | 0.035 |
| S2A_200 | 77307 | 350 | 0.968 | 0.031 | 0.041 | 0.049 | 0.034 |
| S2A_202 | 78066 | 201 | 0.971 | 0.026 | 0.038 | 0.045 | 0.036 |
| S2A_205 | 93719 | 263 | 0.970 | 0.029 | 0.039 | 0.047 | 0.032 |
| S3A_209 | 156273 | 587 | 0.964 | 0.033 | 0.047 | 0.057 | 0.043 |
| S4_176 | 162509 | 405 | 0.959 | 0.036 | 0.054 | 0.065 | 0.053 |
| S4_180 | 171217 | 186 | 0.969 | 0.030 | 0.041 | 0.049 | 0.033 |
| S4_193 | 143227 | 155 | 0.973 | 0.025 | 0.035 | 0.042 | 0.031 |
| S4_197 | 142929 | 194 | 0.972 | 0.029 | 0.036 | 0.043 | 0.026 |
| S4_212 | 158371 | 252 | 0.967 | 0.032 | 0.043 | 0.051 | 0.036 |
| S5_187 | 150179 | 123 | 0.971 | 0.025 | 0.038 | 0.046 | 0.037 |
| S5_192 | 130625 | 84 | 0.975 | 0.022 | 0.033 | 0.040 | 0.032 |
| S5_195 | 116056 | 173 | 0.969 | 0.027 | 0.040 | 0.048 | 0.038 |
| S5_211 | 144835 | 148 | 0.976 | 0.025 | 0.032 | 0.038 | 0.024 |
| S5_240 | 132205 | 150 | 0.970 | 0.028 | 0.039 | 0.047 | 0.036 |
| S6A_161 | 95490 | 111 | 0.970 | 0.024 | 0.039 | 0.046 | 0.042 |
| S6A_163 | 121025 | 124 | 0.973 | 0.022 | 0.035 | 0.042 | 0.037 |
| S6A_171 | 132799 | 59 | 0.975 | 0.022 | 0.032 | 0.039 | 0.031 |
| S6A_172 | 149339 | 21 | 0.978 | 0.017 | 0.028 | 0.034 | 0.029 |
| S6A_242 | 139664 | 99 | 0.974 | 0.021 | 0.033 | 0.040 | 0.035 |
| S6B_162 | 133032 | 90 | 0.974 | 0.022 | 0.033 | 0.040 | 0.033 |
| S6B_167 | 131238 | 18 | 0.976 | 0.019 | 0.031 | 0.037 | 0.033 |
| S6B_168 | 156490 | 36 | 0.976 | 0.020 | 0.031 | 0.038 | 0.032 |
| S6B_169 | 151740 | 28 | 0.978 | 0.017 | 0.027 | 0.033 | 0.029 |
| S6B_241 | 156471 | 87 | 0.972 | 0.026 | 0.037 | 0.044 | 0.032 |
| S7B_173 | 172660 | 327 | 0.966 | 0.031 | 0.044 | 0.054 | 0.039 |
| S7B_174 | 157886 | 282 | 0.975 | 0.021 | 0.032 | 0.039 | 0.032 |
| S7B_175 | 140682 | 128 | 0.979 | 0.016 | 0.027 | 0.033 | 0.030 |
| S7B_184 | 159904 | 56 | 0.975 | 0.026 | 0.031 | 0.041 | 0.022 |
| Anatone | 165676 | 247 | 0.975 | 0.022 | 0.033 | 0.039 | 0.031 |
| Columbia | 144120 | 109 | 0.976 | 0.022 | 0.031 | 0.037 | 0.028 |
| Goldar | 141306 | 148 | 0.978 | 0.021 | 0.028 | 0.033 | 0.023 |
| P-7 | 151944 | 139 | 0.974 | 0.024 | 0.034 | 0.041 | 0.031 |
| Wahluke | 158416 | 161 | 0.973 | 0.023 | 0.034 | 0.041 | 0.033 |
| Whitmar | 68656 | 358 | 0.976 | 0.023 | 0.031 | 0.037 | 0.027 |
